# Effect of La_0.4_Sr_0.6_MnO_3_ Concentration on Physicochemical, Mechanical, and Antibacterial Properties of Babassu Membranes

**DOI:** 10.1021/acsomega.6c04631

**Published:** 2026-08-22

**Authors:** Emanuella de Araújo Carvalho, Vanessa Santana Silva Favacho, Raquel de Melo Barbosa, César Viseras Iborra, Valdeci Bosco dos Santos, Débora dos Santos Tavares, Cristiane Xavier Resende

**Affiliations:** † Postgraduate Program in Materials Science and Engineering, Federal University of Sergipe - UFS, 49107-230 São Cristóvão, SE, Brazil; ‡ Department of Materials Science and Engineering, Federal University of Sergipe - UFS, 49107-230 São Cristóvão, SE, Brazil; § Department of Pharmacy and Pharmaceutical Technology, 16741 University of Granada - UGR, 18071 Granada, Spain; ∥ Raw Materials, Human and Environmental Health, University of Granada - UGR, Associated Unit of the CSIC by the IACT-CSIC, Avda. De Las Palmeras No. 4, 18100 Armilla, Granada, Spain; ⊥ Department of Materials Engineering, 67823 Federal University of Piauí - UFPI, 64049-550 Teresina, PI, Brazil; # Department of Health Education, 701868 Federal University of Sergipe - UFS, 49400-000 Lagarto, SE, Brazil

## Abstract

The treatment of skin injuries related to burns and chronic diseases is a public health challenge. Babassu coconut mesocarp flour has been used in biomedical applications due to its anti-inflammatory and healing properties. Additionally, bioactive substances such as La_0.4_Sr_0.6_MnO_3_ particles can be incorporated into the polymer matrix, conferring magnetic and antibacterial properties. In this work, the effect of La_0.4_Sr_0.6_MnO_3_ concentration (0, 5, and 10%) on babassu membranes containing vitamin C (MNP0, MNP5, and MNP10) was evaluated in terms of their wettability, degradation, permeability, mechanical, magnetic, and antibacterial properties. The higher concentration of manganite in the membranes increased the wettability, the tensile strength (2.9 MPa), the elongation at break (67%), and the stiffness, the latter evidenced by both the elastic modulus (4.2 GPa) and hardness values (0.22 GPa). Additionally, the degradation resistance was also improved. Moreover, an increase in the magnetic moment was observed, reaching approximately 0.10 emu/g for MNP10. Regarding the antibacterial activity of the membranes, the MNP10 exhibited a significantly larger inhibition zone. Therefore, the membrane with 10% (w/w) La_0.4_Sr_0.6_MnO_3_ showed the best performance, suggesting its potential as a wound dressing material.

## Graphical abstract

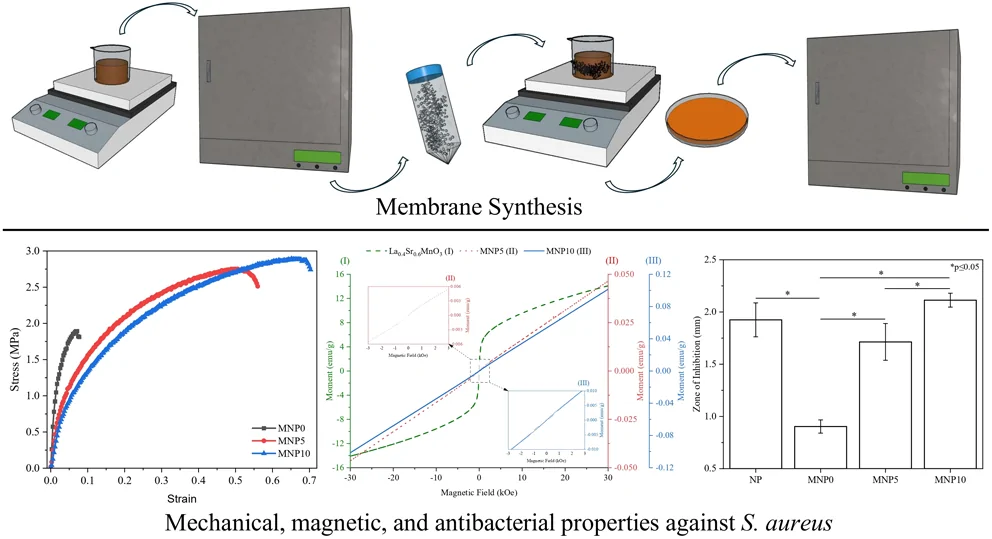

## Introduction

Tissue engineering has focused on developing effective methods for skin repair, particularly in cases of chronic wounds, which are characterized by delayed healing and may result in bacterial infections, excessive loss of water and proteins, and disruptions in the immune system.^1,2^ Conventional dressings, typically composed of gauze, adhesive tape, and cotton, serve only to cover the wound. They are often unable to maintain adequate moisture levels and may leave residues that promote granuloma formation and damage the epithelial flora during frequent changes. Moreover, these dressings lack bioactive properties that could actively support the healing process.^3^

Polymer-based dressings, whether derived from natural or synthetic sources, have been widely used in the treatment of chronic wounds.^4^ A natural polymer abundant and low-cost in Brazil is derived from the flour of the babassu coconut mesocarp. It is composed of over 60% starch, this material is also rich in tannins, anthocyanins, and polysaccharides — compounds with well-established pharmacological properties, including antioxidant, antibacterial, and anti-inflammatory activities — making it a promising candidate for biomedical applications.^5,6^ Essential characteristics of these polymeric dressings include high absorption capacity, adequate water vapor permeability, the ability to maintain a moist environment, antimicrobial properties, good mechanical performance, and the capacity to release bioactive agents.^4,7^ Among the bioactive agents used to support wound healing, both vitamins and magnetic nanoparticles are of particular interest.

Vitamin C, for instance, has been reported in the literature to play a crucial role in the wound healing process, serving as an essential nutrient for collagen production, which promotes cell regeneration.^8^ Additionally, its antioxidant properties enable it to neutralize free radicals — such as superoxide ions, hydroxyl radicals, and singlet oxygen — thereby protecting the skin from inflammatory processes, carcinogens, and other factors that contribute to photoaging.^9,10^ Magnetic nanoparticles, in turn, can accelerate the wound-healing process when exposed to an electromagnetic field and can impart important functionalities to dressings depending on their composition.^11–14^ These functionalities include antibacterial activity, enhanced fluid retention, and improved wettability of the material.^15,16^

Strontium-doped lanthanum Manganite may exhibit magnetic properties, as well as other interesting characteristics in biomedical applications. For instance, a recent antimicrobial study demonstrated that compositions containing La^3+^ ions could be effective in reducing the viability of microorganisms such as *Staphylococcus aureus*. An increase in the concentration of this lanthanide has been shown to significantly inhibit bacterial activity, likely due to the active binding of La^3+^ ions at cell wall sites.^17^ Additionally, nanomaterials conjugated with strontium have exhibited antimicrobial properties and can be employed in targeted drug delivery systems. Sr^2+^ ions can also remain in the body for extended periods, contributing to a sustained immune response and making it a promising agent for immunotherapy applications.^18^

In addition, the proposed membrane system also presents potential relevance from a sustainability perspective. The use of biopolymeric and renewable materials aligns with emerging concepts in green membrane materials and processes, particularly by reducing dependence on fossil-derived materials and valorizing natural resources. Recent advances in membrane science have increasingly explored renewable biobased feedstocks and biomass-derived materials as promising routes toward more sustainable membrane technologies.^19–22^ Furthermore, the use of babassu-derived components may contribute to the valorization of agroextractive residues generated in abundance, especially in regions such as Maranhão, Brazil, where large quantities of babassu coconut residues are produced annually.^7,23^ Thus, the proposed material shows potential alignment with the principles of sustainable membrane development related to responsible consumption, waste reduction, and sustainable material development.^24^

In this study, the effect of La_0.4_Sr_0.6_MnO_3_ concentration on babassu coconut mesocarp flour-based membranes containing vitamin C was investigated with respect to morphology, topography, and thermal stability, using Scanning Electron Microscopy with Energy Dispersive Spectroscopy (SEM-EDS), Atomic Force Microscopy (AFM), and Thermogravimetric Analysis (TGA/DTG), respectively. Furthermore, the membranes were also evaluated regarding degradation in phosphate-buffered saline (PBS) solution, wettability, water swelling capacity, permeability, mechanical behavior, magnetic response, and antibacterial activity.

## Materials and Methods

### Materials

Babassu coconut mesocarp flour (Babcoall Inc. do Brasil LTDA), ultrapure water, distilled water, glycerin (99.5% purity, Neon), l-ascorbic acid (99.7% purity, Neon), ethylene glycol (99.5% purity, Dinâmica), anhydrous citric acid (99.86% purity, Neon), manganese­(II) nitrate tetrahydrate (99.5% purity, Sigma-Aldrich), lanthanum nitrate (99.9% purity, Êxodo Científica), and strontium nitrate (99.0% purity, Sigma-Aldrich) were used in the preparation of the membranes and the synthesis of the Manganite. Formamide (99.5% purity, Sigma-Aldrich) was used for contact angle measurements.

## Synthesis of the Manganite

Strontium-doped lanthanum Manganite (La_0.4_Sr_0.6_MnO_3_) was synthesized using the Pechini method, with citric acid as the chelating agent and ethylene glycol as the polymerizing agent, in a 60:40 mass ratio. The molar ratio of citric acid to total metal cations was 3:1. The procedure was adapted from Rabelo et al.^25^ Initially, citric acid was dissolved in distilled water under magnetic stirring at 60 °C. Reagent solutions were then added at 30 min intervals in the following order: manganese­(II) nitrate and lanthanum­(III) nitrate. After increasing the temperature to 70 °C, strontium nitrate was added, followed 30 min later by ethylene glycol. The temperature was subsequently raised to 90 °C, and the mixture was maintained under constant magnetic stirring until a polymeric gel was formed. The resulting material was precalcined at 300 °C for 4 h, ground into a fine powder, and then subjected to thermal treatment at 900 °C for 4 h, with a heating rate of 10 °C/min.

## Membrane Synthesis

After milling the babassu flour to a 325-mesh particle size, membranes were prepared using the casting method. A 4% (w/v) babassu flour (BF) solution was prepared in 20 mL of ultrapure water, with 30% (w/w) glycerol added as a plasticizer. The mixture was magnetically stirred at room temperature for 30 min and then heated to 90 °C for an additional 30 min under stirring. After cooling to room temperature, 50% (w/w) ascorbic acid was added to all polymer solutions, while varying the concentration of La_0.4_Sr_0.6_MnO_3_ at 0%, 5%, and 10% (w/w). The reduction at room temperature was designed to preserve the bioactive compound and prevent the degradation of ascorbic acid associated with elevated temperatures. The solutions were stirred for an additional 15 min before being cast into Petri dishes and dried in an oven at 37 °C. The membranes were labeled MNP0, MNP5, and MNP10, corresponding to Manganite concentrations of 0%, 5%, and 10%, respectively.

## Characterization

### X-ray Diffraction (XRD)

The Manganite was characterized by X-ray diffraction (XRD) using a Shimadzu LabX XRD-6000 diffractometer with Cu Kα radiation, operating at 40 kV and 30 mA in continuous scan mode. Data were collected with a step size of 1°/min over a 2θ range of 10° to 80°. The crystalline phases present in the sample were identified using XPert HighScore Plus software, and the phase quantification and crystallite size were determined using the MAUD 2.996 software.

## Thermogravimetric Analysis (TGA/DTG)

Thermogravimetric analysis (TGA) and differential thermogravimetric analysis (DTG) were performed to evaluate the thermal behavior of the membranes using a Netzsch STA 449 F3 Simultaneous Thermal Analyzer. Mass loss and thermal events were recorded from 25 to 600 °C at a heating rate of 10 °C/min. For comparison, the analyses were also conducted on babassu flour (BF) and ascorbic acid (AA).

## Scanning Electron Microscopy and Energy Dispersive Spectroscopy (SEM-EDS)

The morphology and elemental composition (qualitative and semiquantitative) of the membrane surfaces were analyzed by scanning electron microscopy (SEM, JSM-5700) equipped with energy-dispersive spectroscopy (EDS). Additionally, the morphology of the La_0.4_Sr_0.6_MnO_3_ ceramic powder was examined, and the average particle size was estimated using ImageJ software. To enable electron microscopy imaging, the membranes and Manganite powder were sputter-coated with gold for 60 s.

## Atomic Force Microscopy (AFM)

Sample images were acquired using a Park Systems NX20 atomic force microscope (AFM) operating in tapping mode to analyze the morphology of the membranes. Image processing was performed using Park Systems’ XEI software.

## Fourier Transform Infrared Spectroscopy (FTIR)

Functional groups present in the membranes were identified using Attenuated Total Reflectance Fourier Transform Infrared Spectroscopy (ATR-FTIR) over the spectral range of 4000 to 600 cm^–1^, recorded on a PerkinElmer Spectrum ES spectrophotometer.

## Determination of Contact Angle and Calculation of Surface Tension

Ultrapure water was used as the solvent to evaluate the wettability of the membranes, and formamide was employed to assist in calculating the surface tension components. For this purpose, each membrane was placed individually on a smooth glass plate, and a drop of liquid was deposited on its surface. The contact angle formed at the interface between the membrane surface and the liquid was measured using a digital microscope (2.0 MP USB Zoom Camera, 1600× magnification) and analyzed with ImageJ software.

Based on the measured contact angles with ultrapure water and formamide, the approximate surface tension components of the samples were calculated using eq 1, following the Kaelble method.^26^

1 $$\gamma_{L}\times \left[1+\cos\left(\theta \right)\right]=2{\left({\gamma_{S}}^{d}\times {\gamma_{L}}^{d}\right)}^{1/2}+2{\left({\gamma_{S}}^{p}\times {\gamma_{S}}^{p}\right)}^{1/2}$$

In this equation, θ represents the contact angle formed between the liquid and the solid surface; γ_S_^d^ and γ_S_^p^ are the dispersive and polar components of the solid’s surface tension, respectively, whose sum corresponds to the total surface free energy of the solid (γ_S_). The surface tension (γ_L_) of water and formamide is 72.8 and 58.0 mJ·m^–2^, respectively. For water, the dispersive (γ_L_^d^) and polar (γ_L_^p^) components are 21.8 and 51.0 mJ·m^–2^, respectively, while for formamide the γ_L_^d^ is 39.0 mJ·m^–2^ and γ_L_^p^ is 19.0 mJ·m^–2^.^27^

Thus, although formamide is also a polar liquid, it has different surface tension characteristics compared to water and can provide useful contact angle data due to its sufficiently high surface tension, which prevents it from spreading on the surface.^28^

## Swelling Degree

The hydration degree of the membranes was evaluated to assess their ability to retain liquid while remaining moist. To this end, membrane samples (1.0 cm^2^) were weighed using an analytical balance to obtain the dry sample weight (W_i_). Each membrane was then individually immersed in 5 mL of ultrapure water and maintained at 37 °C for predetermined time intervals (0.25, 0.5, 1, 2, 4, 8, 16, 24, and 48 h). After each interval, excess liquid was removed from the membranes using filter paper, and the samples were weighed again (W_f_) to determine the mass variation. The degree of swelling was calculated using eq 2, in accordance with ASTM D644-99.^29^

2 $$\mathrm{swelling}\;\left(\%\right)=\frac{W_{f}-W_{i}}{W_{i}}\times 100$$

## *In Vitro* Degradation

To analyze hydrolytic degradation, samples of MNP0, MNP5, and MNP10 (each with an area of 1 cm^2^) were weighed (*W*_0_) and placed into Falcon tubes containing 5 mL of phosphate-buffered saline (PBS) at pH 7.4. The samples were then incubated at 37 °C for 8, 16, 24, and 32 days. At each predetermined time point, the membranes were washed with ultrapure water, dried at 40 °C, and then reweighed (*W_t_*). The mass loss of each sample was calculated using eq 3.

3 $$w\mathrm{eight loss}\;\left(\%\right)=\frac{W_{0}-W_{t}}{W_{t}}\times 100$$

## Water Vapor Permeability and Transmission Rate

The water vapor permeability (WVP) of the membranes was determined according to ASTM E96/E96M-16.^30^ For this purpose, the membranes were fixed onto acrylic cells maintained at 22 ± 2 °C, with silica gel placed at the bottom of the cells to maintain a relative humidity of 0% (*R*_2_). These cells were then placed inside a hermetically sealed desiccator containing a saturated NaCl solution at the bottom, which maintained the internal environment at a relative humidity of 69% (*R*_1_). By monitoring the increase in total cell mass at time of 1, 2, 3, 4, 24, 48, 72, and 96 h, the WVP was calculated using eq 4. In this equation, φ represents the slope of the line obtained from the increase in mass over time (g/h), δ is the membrane thickness (mm), *A* is the area of the internal opening of the cell covered by the membrane (m^2^), and *S* is the saturation vapor pressure at the test temperature (2650 Pa at 22 °C).

4 $$\mathrm{WVP}=\left(\frac{\varphi \times \delta }{A\times S\times \left(R_{1}-R_{2}\right)}\right)$$

The water vapor transmission rate (WVT) was calculated using eq 5.

$$\mathrm{WVT}=(\frac{\mathrm{WVP}}{\delta })\times S\times \left(R_{1}-R_{2}\right)$$ 5

## Mechanical Properties Assessment by Tensile Testing

Membrane samples were cut into rectangular strips with fractured sections of 90 × 25 × 0.18 mm. Tensile testing was carried out using an Instron 3367 universal testing machine equipped with Instron 2712–019 pneumatic grips and an initial grip separation of 35 mm. The membranes were stretched at a constant rate of 1 mm/s until fracture, in accordance with ASTM D882.^31^

## Mechanical Properties Assessment by Nanoindentation

Nanoindentation tests were performed using a Bruker Hysitron TI Premier system coupled to a Park Systems NX20 atomic force microscope. For each sample, 16 indentations were performed in a 4 × 4 matrix, with 15 μm spacing between indentations in both horizontal and vertical directions. A load ranging from 0 to 1500 μN was applied.

## Magnetic Susceptibility Analysis

The magnetic susceptibility of the ceramic powder La_0.4_Sr_0.6_MnO_3_ and of the membranes containing magnetic nanoparticles (MNP5 and MNP10) was measured as a function of the applied magnetic field at a temperature of 300 K, using a PPMS DynaCool magnetometer.

## Antibacterial Susceptibility Test

The antibacterial activity of strontium-doped lanthanum Manganite and the composite membranes was evaluated using the disk diffusion method against the Gram-positive strain *Staphylococcus aureus* (NCTC 12973), provided by the Central Public Health Laboratory of Sergipe (LACEN-SE). *S. aureus* was inoculated in a broth containing meat extract (1 g·L^–1^), yeast extract (2 g·L^–1^), peptone (5 g·L^–1^), and NaCl (5 g·L^–1^), and incubated at 37 °C for 24 h. After incubation, the broth was seeded onto nutrient agar plates using sterile swabs. Strontium-doped lanthanum Manganite tablets (6 mm diameter) and membrane samples cut into disks of the same dimensions were placed onto the inoculated agar plates. The plates were incubated at 37 °C for 48 h in a bacteriological incubator. After this period, the presence or absence of inhibition halos around the samples was evaluated.

## Statistical Analysis

All experiments were conducted in triplicate, and the results were presented as mean ± standard deviation. One-way analysis of variance (ANOVA) followed by Tukey’s post hoc test (*p* ≤ 0.05) was used to analyze the EDS data, surface roughness, contact angle, surface tension, *in vitro* degradation, permeability, water vapor transmission rate, antibacterial activity, and mechanical properties (tensile and nanoindentation).

## Results and Discussion

### X-ray Diffraction (XRD)

Figure 1 presents the X-ray diffraction (XRD) pattern of the sample after calcination. The crystalline phase was identified as La_0.4_Sr_0.6_MnO_3_, with no evidence of secondary or spurious phases, as confirmed by Rietveld refinement (GoF = 1.32 and Rwp = 14.97%).

**Figure 1:**
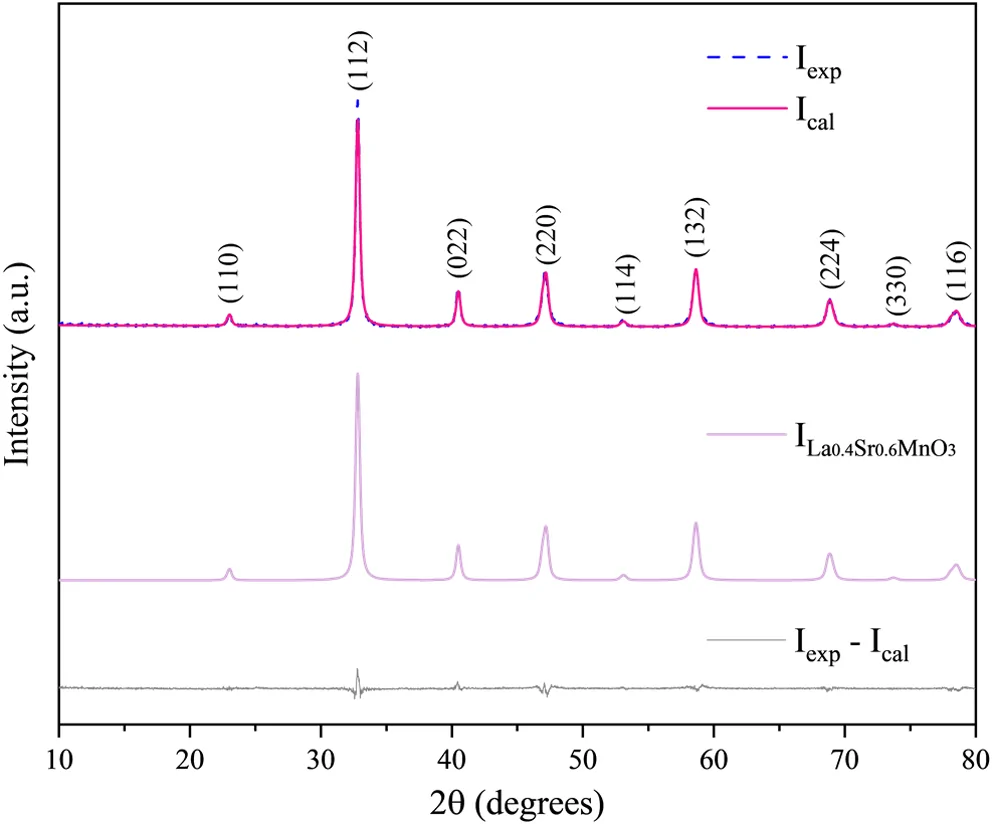
XRD pattern of La_0.4_Sr_0.6_MnO_3_ powder.

According to the Rietveld refinement, the La_0.4_Sr_0.6_MnO_3_ phase (ICSD file no. 236944) crystallizes in a tetragonal perovskite structure (*I4/mcm* space group) with lattice parameters *a* = *b* = 5.4420 Å and *c* = 7.7397 Å. This phase exhibited prominent X-ray diffraction peaks at 23.00, 32.91, 40.53, 46.98, 53.37, 58.54, 68.99, 73.42, and 78.93° 2θ, corresponding to the crystallographic planes (110), (112), (022), (220), (114), (132), (224), (330), and (116), respectively. Furthermore, the average crystallite size, calculated from Rietveld refinement, was 39 ± 0.1 nm, indicating that the synthesized ceramic powder predominantly consists of nanoparticles. For comparison, Ibrahim et al.^32^ synthesized nanoparticles with the exact stoichiometry and calcined them at 900 °C for 5 h, resulting in a crystallite size of 63 nm. Therefore, the nanoparticles produced in this study exhibited a smaller crystallite size than those previously reported in the literature.

## Thermogravimetric Analysis (TGA/DTG)

Babassu flour (FB) exhibits two main stages of degradation, as demonstrated by the results of thermogravimetric analysis (Figure 2a). The first mass loss occurred around 100 °C and was attributed to the elimination of physically adsorbed water within the FB structure. The second stage corresponded to the thermal degradation of FB, with a maximum degradation rate observed at approximately 304.73 °C. By the end of the analysis, at 600 °C, approximately 80% of the sample’s initial mass had been lost.^33^ As for ascorbic acid (AA), degradation began at approximately 188 °C, with the highest mass loss rate occurring at 227.42 °C. This peak was followed by two additional, less pronounced degradation stages within the temperature range of 250–600 °C.^34^ At 600 °C, approximately 80% of the original mass was lost, with the remaining ∼20% attributed to carbonized residue.

**Figure 2:**
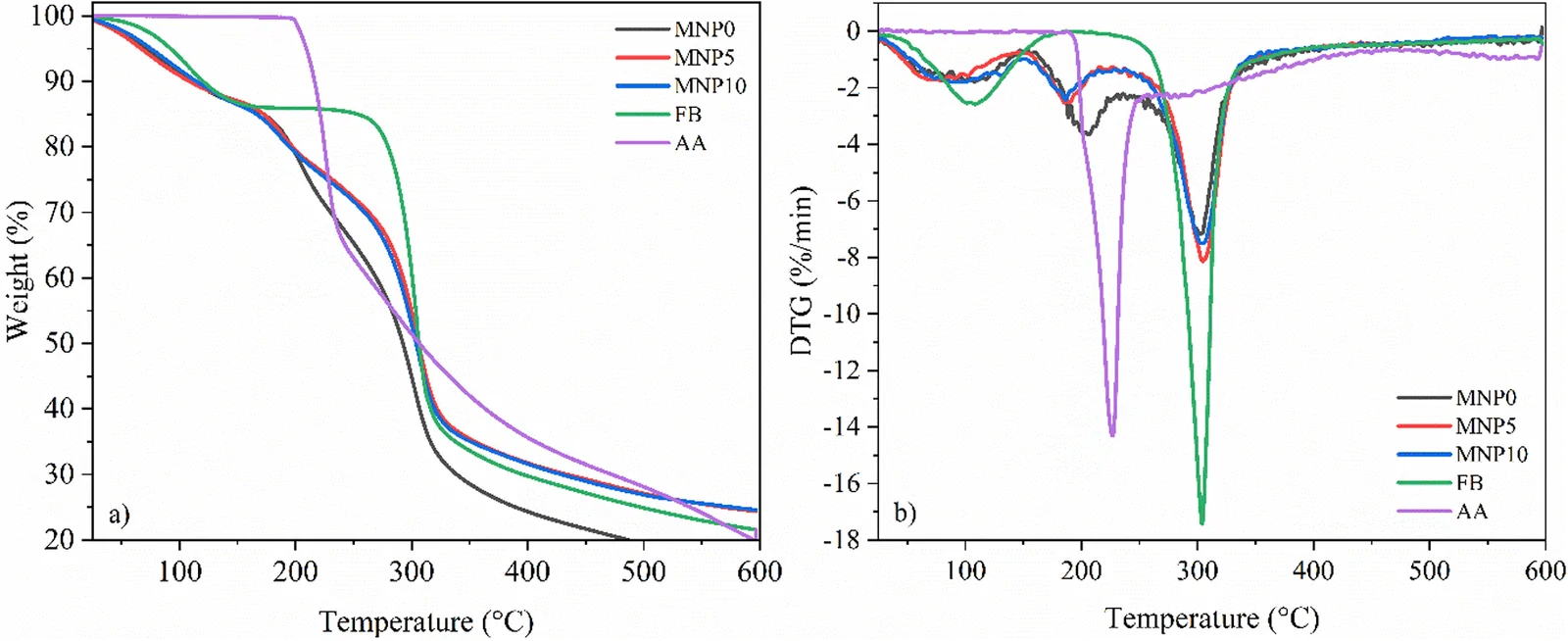
(a) TGA and (b) DTG curves of babassu flour (FB), ascorbic acid (AA), and membrane samples (MNP0, MNP5, and MNP10).

Regarding the thermal behavior of the membranes, three main mass loss events were observed up to approximately 350 °C across all studied compositions. These events corresponded to the elimination of water, the maximum degradation of ascorbic acid (AA), and the degradation of babassu flour components, respectively. The MNP5 and MNP10 membranes exhibited very similar total mass losses, 75.63 and 75.44%, respectively, indicating that the incorporation of 5 and 10% (w/w) of Manganite reduced the overall mass loss by nearly 10% compared to the MNP0 membrane.^35^ This result suggests an effective interaction between the inorganic filler (Manganite) and the polymer matrix (babassu flour), indicating good dispersion of the filler particles within the composite structure.^36^

## Scanning Electron Microscopy and Energy Dispersive Spectroscopy (SEM-EDS)

Given that the membrane is intended for direct contact with the skin, its morphology is a critical property for predicting its behavior and interactions within the human body. Ideally, the membrane should be permeable, enabling fluid flow through its porous structure, while the pore dimensions should be sufficient to inhibit the penetration of bacterial cells.^37^ Figure 3 presents the SEM micrographs of the La_0.4_Sr_0.6_MnO_3_ powder and the surface morphology of the obtained membranes.

**Figure 3:**
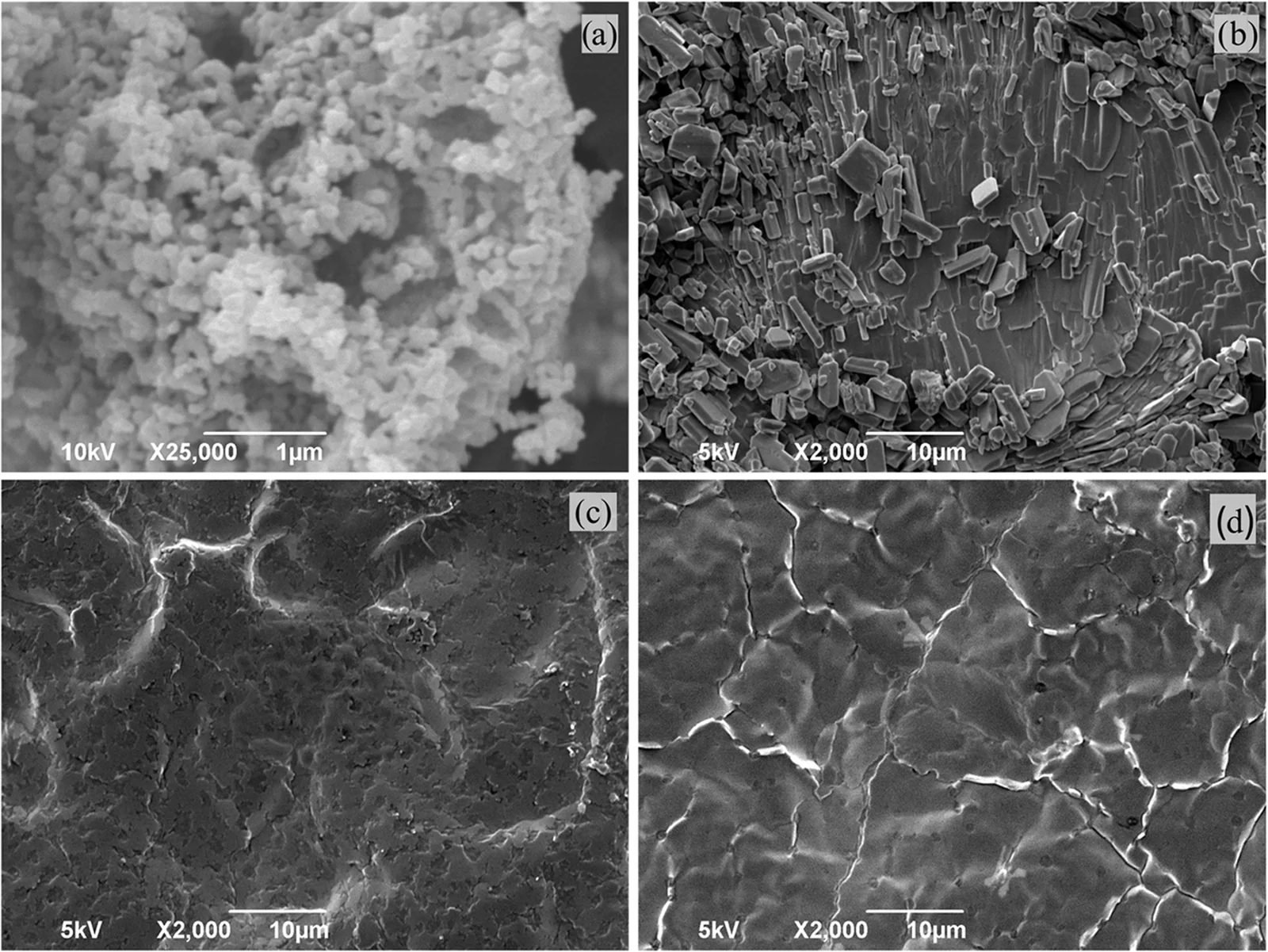
SEM micrographs of: (a) La_0.4_Sr_0.6_MnO_3_ powder, (b) MNP0, (c) MNP5, and (d) MNP10 membranes.

The micrographs reveal that the La_0.4_Sr_0.6_MnO_3_ nanoparticles exhibited a spherical morphology, with an estimated average size of 79 ± 14 nm. Additionally, vitamin C crystals are observed on the surface of MNP0 (Figure 3b), whereas the surfaces of MNP5 and MNP10 membranes (Figure 3c,d) became progressively more homogeneous with increasing Manganite nanoparticle content. This homogeneity was attributed to the improved dispersion of nanoparticles within the polymeric matrix, as evidenced by thermogravimetric analysis, which suggests enhanced interaction between the nanoparticles and the biopolymer. Such interactions optimize nanoparticle anchoring sites on the membrane surface, facilitating increased liquid flow and reducing scale formation.^38^ Moreover, a higher nanoparticle concentration increased the viscosity of the synthesis solution, leading to membranes with denser surfaces.^39^ Consequently, the nanocomposite membranes exhibited smoother and more compact surfaces. These characteristics may result in a more effective permeability barrier and allow greater water flow over the surface, as observed by Jeong et al.^40^ and Alhoshan et al.^41^

Energy-dispersive X-ray spectroscopy (EDS) analysis was also conducted to characterize the elemental composition on the membrane surfaces semiquantitatively. The atomic percentages of the identified elements are presented in Figure 4.

**Figure 4:**
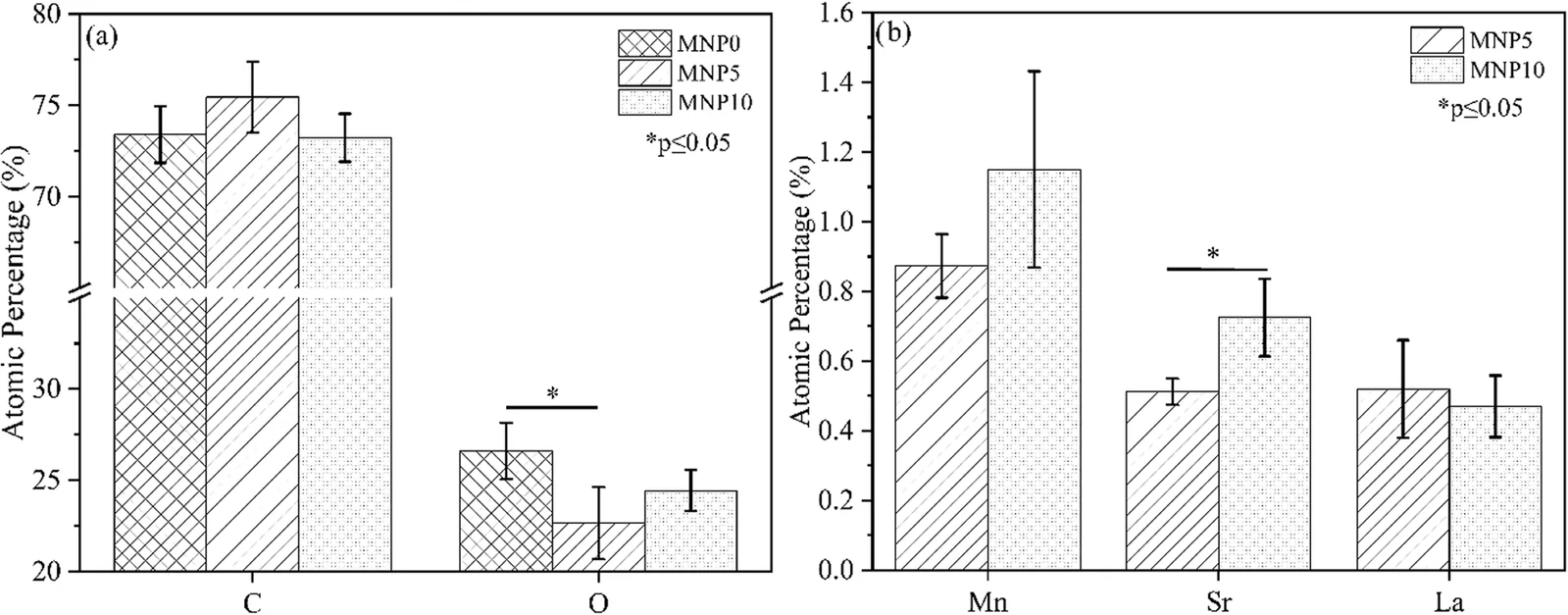
Atomic percentages of elements present in the membranes: (a) carbon and oxygen; (b) manganese, strontium, and lanthanum.

Since the membranes are primarily composed of organic compounds, carbon (C) and oxygen (O) were the main elements detected, with atomic percentages of 75 ± 2 and 27 ± 2%, respectively, as shown in Figure 4a. It is important to note that the membranes were mounted on carbon tape for adhesion to the sample holder; therefore, a portion of the detected carbon (75 ± 2%) may originate from this substrate. Upon incorporation of Manganite nanoparticles into the polymer matrix of MNP5 and MNP10, the presence of strontium (Sr), manganese (Mn), and lanthanum (La) was detected, confirming the successful deposition of Manganite particles on the membrane surfaces. These elements were not detected in MNP0, consistent with its composition.

## Atomic Force Microscopy (AFM)

The increase in surface homogeneity of the membranes with rising concentrations of Manganite nanoparticles was evident from the AFM images presented in Figure 5.

**Figure 5:**
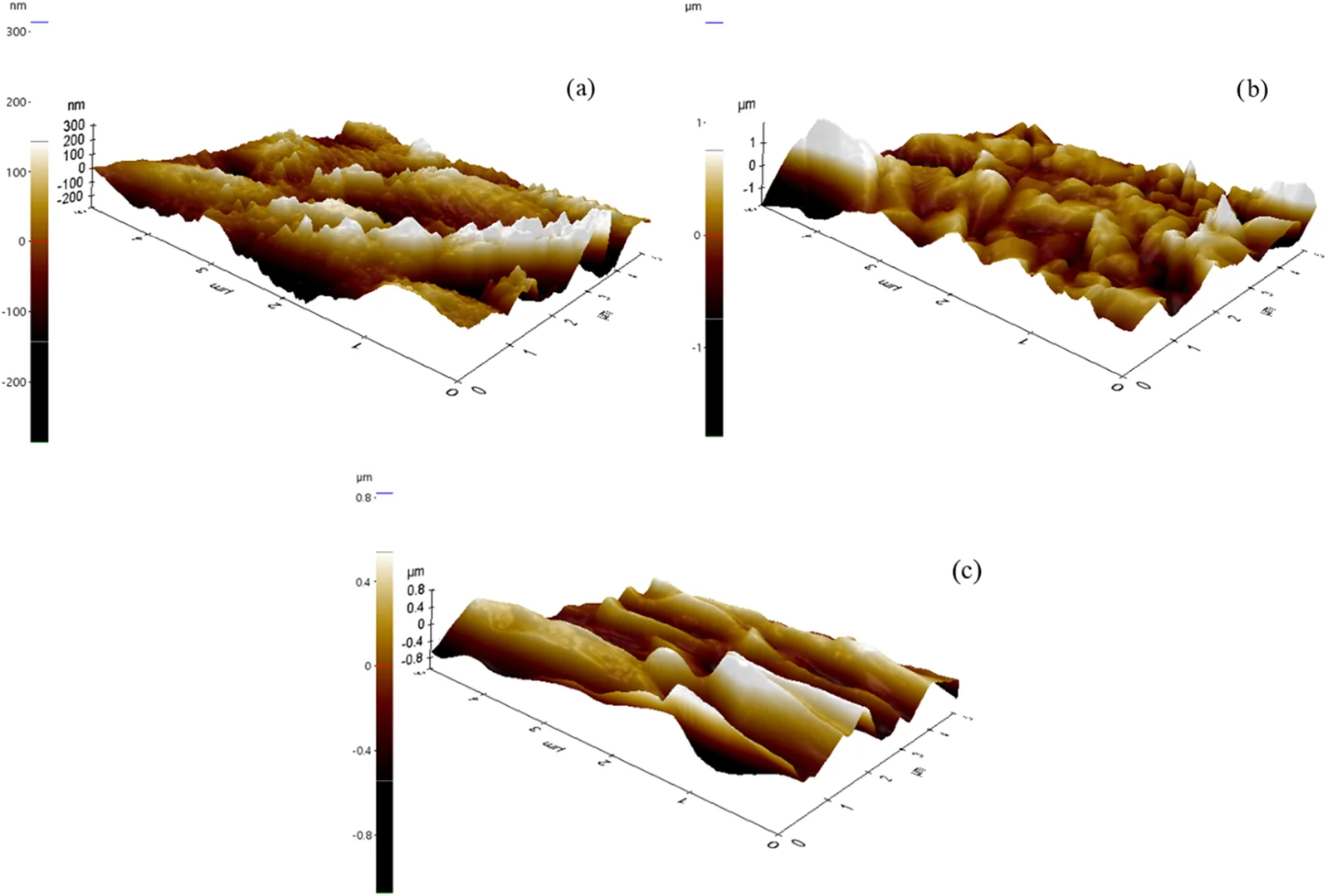
AFM images of membrane surfaces in 5 × 5 μm areas: (a) MNP0; (b) MNP5; (c) MNP10.

The surface topography of MNP5 and MNP10 differed markedly from that of MNP0 (Figure 5), as the latter exhibited visibly higher surface roughness due to the presence of vitamin C crystals dispersed across the surface, corroborating the SEM micrographs presented in Figure 3. However, quantitative analysis (Table 1) of the mean roughness (*R*_a_), root-mean-square roughness (*R*_q_), and surface area (*A*_s_) revealed that MNP5 and MNP10 actually displayed significantly higher roughness values compared to MNP0. Moreover, MNP5 showed significantly higher roughness compared to MNP10.

**Table 1:** Roughness and Surface Area Data of the Membranes[Table-fn t1fn1]

**sample**	***R*_a_ (nm)**	***R*_q_ (nm)**	***A*_s_ (μm** ^**2**^ **)**
**MNP0**	33 ± 3^a^	45 ± 4^a^	28.9 ± 0.5^a^
**MNP5**	153 ± 11^b^	206 ± 15^b^	61 ± 10^b^
**MNP10**	72.6 ± 0.9^c^	101 ± 2^c^	34 ± 2^a^

d Means followed by different letters within the same column were significantly different according to Tukey’s test at a 5% significance level (*p* ≤ 0.05).

The absence of Manganite nanoparticles in MNP0 indicated that the microscopically visible surface roughness was predominantly governed by vitamin C crystals, which remained uniformly distributed even at the nanoscale, thereby preventing localized accumulations that would create sharp peaks. In contrast, the addition of 5% (w/w) Manganite nanoparticles in MNP5 covered the vitamin C crystals, smoothing their surface coverage but causing nonuniform agglomerations of nanoparticles on the membrane surface. This led to the formation of isolated nanoparticle peaks at the microscale, thereby increasing the roughness and surface area measurements.

Compared to MNP5, increasing the nanoparticle concentration to 10% (w/w) in the MNP10 membrane resulted in a more uniform distribution of the particles on the surface, which contributed to a reduction in both surface roughness and surface area. This effect can be attributed to the increased viscosity of the synthesis solution caused by the higher nanoparticle content. The elevated viscosity facilitates a broader and more uniform occupation of the polymer matrix and reduces the mobility of the dispersed nanoparticles. Consequently, the tendency for localized aggregation is diminished, promoting a more homogeneous nanoparticle distribution throughout the membrane.^42^ Similar observations have been reported by Alhoshan et al.,^41^ Ahmed et al.,^43^ and Ahmed et al.^44^

## Fourier Transform Infrared Spectroscopy (FTIR)

The FTIR analysis was conducted to identify the functional groups present in the membranes. The obtained spectra are shown in Figure 6.

**Figure 6:**
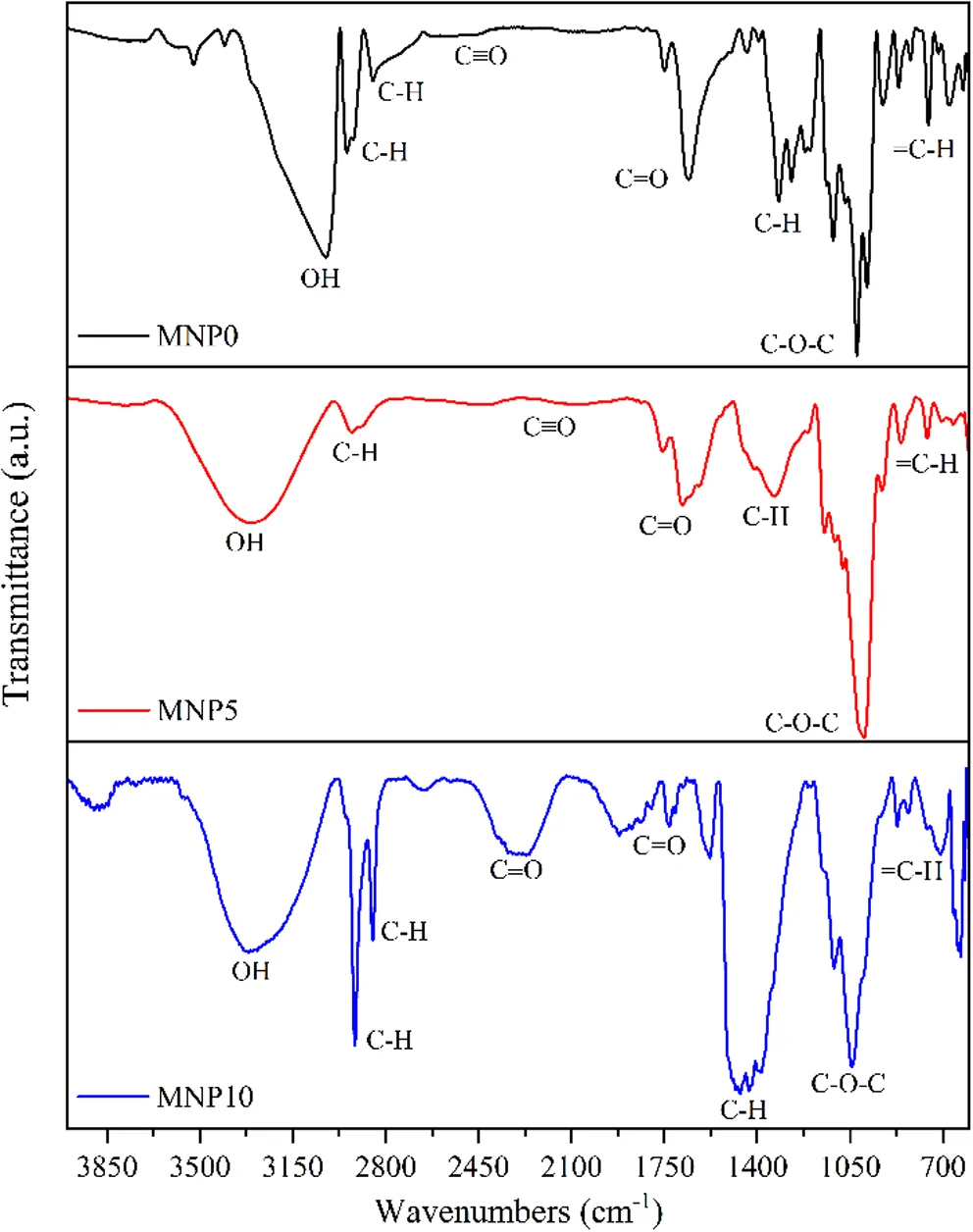
FTIR spectra of the membranes.

The FTIR spectra of all membranes, regardless of composition, exhibited broad bands between 3000 and 3500 cm^–1^, which are attributed to the stretching vibrations of hydroxyl (−OH) groups present in the biopolymer.^45,46^ These interactions between the hydroxyl groups of babassu flour and glycerol, along with hydrogen bonds formed with Manganite particles, resulted in a polymeric complex with an amorphous structure. This complex reduces the lengths of intra- and intermolecular hydrogen bonds, thereby increasing the density and rigidity of the membranes.^35^

Among the compounds that make up the babassu coconut mesocarp, the most prominent are carbohydrates, such as starch and cellulose, as well as proteins, pectin, and lipids.^47^ Based on this principle, characteristic bands appeared at 2918 and 2848 cm^–1^ in MNP0 and MNP10, and at 2888 and 2733 cm^–1^ in MNP5. These bands correspond to C–H stretching vibrations commonly found in proteins, starch, and pectin components of the mesocarp flour.^48,49^ The peaks observed at 1320, 1334, and 1464 cm^–1^ for MNP0, MNP5, and MNP10, respectively, are indicative of C–H bending vibrations typical of cellulose.^50^ Furthermore, absorptions in the 950 to 1200 cm^–1^ range, characteristic of C–O–C stretching vibrations, confirmed the presence of carbohydrates, specifically starch, in the samples.^51,52^ The bands between 856 and 702 cm^–1^ can be assigned to =C–H deformation vibrations of monosubstituted aromatic rings, attributed to the lignin fraction in babassu flour.^53^

Bands observed between 1560 and 1760 cm^–1^ correspond to the characteristic stretching vibrations of carbonyl groups (C=O),^52,54^ while those around 2600 and 2100 cm^–1^ can be attributed to the C≡C functional groups present in the cellulose structure^55^ across all membrane compositions.

The spectral analysis further reveals that MNP5 exhibited greater intensity in the bands corresponding to the identified functional groups compared to MNP0, whereas MNP10 showed a decrease in band intensity. This phenomenon may be explained by the formation of nanoparticle clusters in MNP5, which, due to incomplete dispersion within the polymer matrix, create regions with a more porous microstructure and interfaces that enhance light interaction with exposed functional groups, resulting in increased transmission. Conversely, in MNP10, nanoparticles were more uniformly distributed and deeply embedded in the matrix, with reduced agglomerate formation and produced a denser structure that limits light permeability, thereby decreasing transmittance. Additionally, this increased density may restrict the mobility of functional groups, diminishing the vibrational modes responsible for light transmission.^56^

It was also noted that the C–H bands in MNP10 were more pronounced than in the other samples, a behavior not observed in MNP0 and MNP5. This difference may result from a structural rearrangement of polymer chains due to the more homogeneous dispersion of nanoparticles in MNP10, which increases the exposure of methyl and methylene (C–H) groups on the membrane surface. Simultaneously, polar groups such as – OH and C–O–C may become more encapsulated or confined within microenvironments formed by nanoparticle–polymer interactions, reducing their radiation exposure and, consequently, increasing the relative prominence of C–H bands.^57^

## Determination of Contact Angle and Calculation of Surface Tension

The contact angles between the membrane surfaces and ultrapure water, along with the surface tensions (γS), are presented in Figure 7.

**Figure 7:**
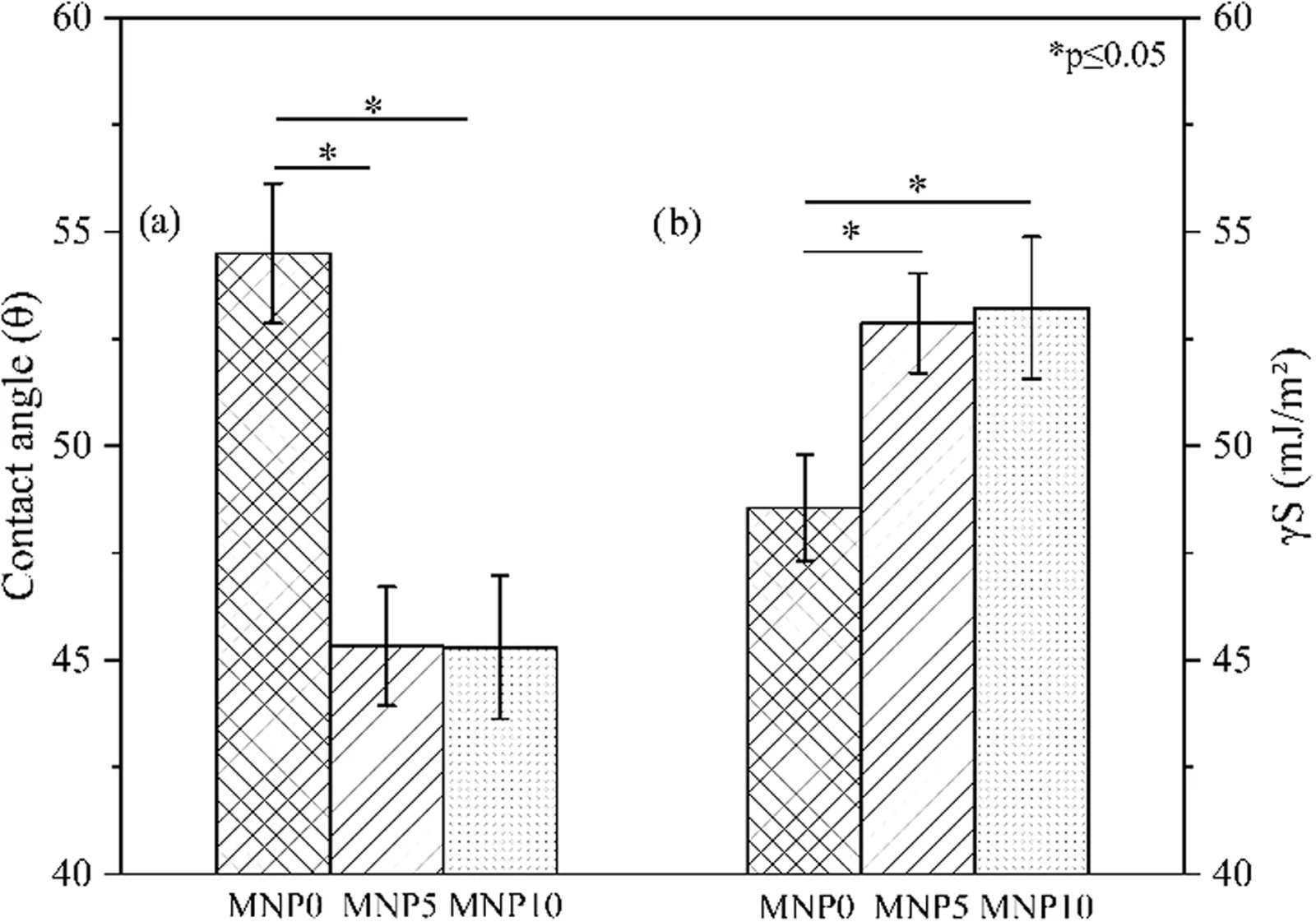
Average (a) contact angles (measured using ultrapure water) and (b) surface tension of the membranes.

Besides starch, the babassu mesocarp composition includes hemicellulose, cellulose, and lignins — components typical of natural fibers with polar characteristics^58^ — in addition to glycerol incorporated into the membranes. These constituents facilitate interaction with water, ensuring the material’s hydrophilicity. Consequently, all membrane compositions exhibited hydrophilic behavior, with contact angles below 90°.

Moreover, the addition of Manganite nanoparticles to the membranes caused a statistically significant decrease in the contact angle between the surface and water. Specifically, MNP5 and MNP10 showed increased hydrophilicity compared to MNP0. This effect can be attributed to the polar nature of Manganite^59^ and its high specific surface area, which enhances the interaction area with the liquid and improves membrane wettability.^60^ Furthermore, AFM data demonstrated that the incorporation of nanoparticles increased the surface roughness of the membranes, resulting in a larger effective surface area; consequently, the number of sites available for interactions increased at the interface between the membrane and the surrounding medium. As particle size decreases, the surface area-to-volume ratio increases, leading to a higher proportion of atoms residing at the particle surface compared to those in the core.^61^ According to Wenzel’s wettability model, increased surface roughness amplifies the intrinsic wettability behavior of a material, making hydrophobic surfaces (θ > 90°) more hydrophobic and hydrophilic surfaces (θ < 90°) more hydrophilic. Since the membranes exhibited contact angles less than 90°, this phenomenon further contributes to the enhanced wettability observed with higher Manganite content.^62^

The high surface-to-volume ratio of nanoparticles results in elevated surface energies, which can promote particle aggregation or clustering in physiological environments and other fluids.^63^ This behavior was also reflected in the observed increase in the surface tension of MNP5 and MNP10 membranes compared to MNP0, following the same trend as the contact angle measurements. These findings indicate that the incorporation of Manganite nanoparticles enhanced the membranes’ interaction with body fluids released from wounded skin.

## Swelling Degree

Water absorption is a critical parameter in determining the suitability of wound dressings,^64^ as effective dressings must possess adequate swelling capacity.^65^ They should be capable not only of absorbing exudates over extended periods but also of transporting various substances such as nutrients, drugs, cells, or genes.^64^ Figure 8 presents the swelling behavior of the samples in water at 37 °C over time.

**Figure 8:**
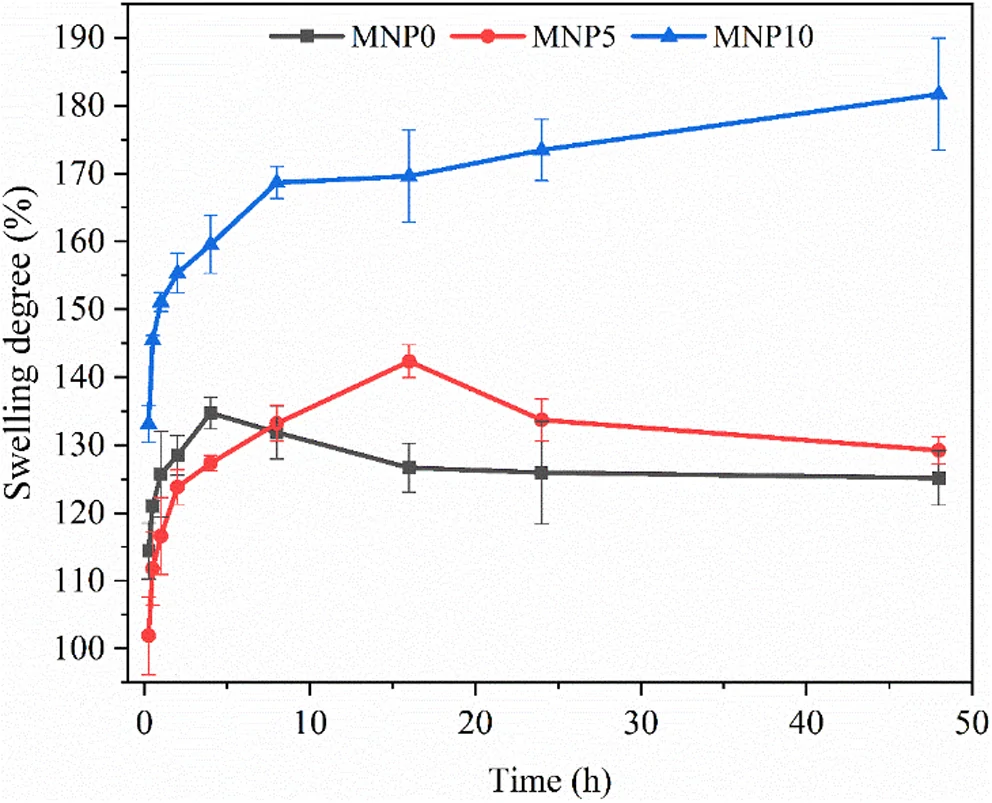
Swelling degree of the membranes.

Analysis of the swelling behavior reveals that MNP0 exhibited an initial water uptake of 114.38% after 15 min, reaching a peak of 134.70% at 4 h. However, this was followed by a decline to 125.12% at 48 h, indicating high initial absorption but limited long-term retention. MNP5 absorbed 101.89% of water at 15 min and reached its maximum swelling (142.32%) at 16 h. Subsequently, a decrease in water retention was observed, suggesting some instability. Nevertheless, MNP5 showed improved overall absorption performance compared to MNP0, particularly over extended periods.

Among the tested membranes, MNP10 demonstrated the most favorable and stable behavior. It showed a consistent increase in swelling across all measured time points, starting at 133.07% after 15 min and reaching 181.66% after 48 h. This indicates that the incorporation of 10% (w/w) Manganite nanoparticles significantly enhanced the membrane’s swelling capacity and long-term stability in aqueous environments.

These phenomena can be attributed to the degradation of vitamin C during the swelling test, which limits the water retention capacity of membranes with absent or low concentrations of nanoparticles. Upon oxidation, ascorbic acid loses electrons and hydrogen atoms, forming dehydroascorbic acid (C_6_H_6_O_6_), which subsequently undergoes hydrolysis to produce 2,3-diketogulonic acid.^66^ The degradation products are less hydrophilic than the original ascorbic acid, resulting in reduced interaction with water and, consequently, lower retention over time.^67^

In contrast, the superior performance of MNP10 suggests that vitamin C may be encapsulated within the membrane’s pore structure, acting as a protected reservoir and limiting exposure to oxidative agents such as oxygen. During membrane synthesis, as water is evaporated from the precursor solution, vitamin C is likely to become entrapped within the forming pores. Simultaneously, the added Manganite nanoparticles preferentially distribute over these regions, decreasing pore size and contributing to a smoother, denser surface. This hypothesis is supported by the FTIR spectra, as well as morphological analyses observed in the SEM and AFM results.

## *In vitro* Degradation

As shown in Figure 9, the membranes exhibited a time-dependent degradation profile, with mass loss increasing progressively across all samples throughout the evaluation period.^68^ In this context, the observed mass loss should be understood as the overall mass reduction behavior of the membranes in an aqueous medium, involving both polymer relaxation and the leaching of soluble compounds, such as glycerol and ascorbic acid. Although vitamin C was incorporated only after the polymer solution had cooled to room temperature to prevent its degradation, no analytical assessment of its stability or retention was performed; this represents an interesting aspect for investigation in future studies.

**Figure 9:**
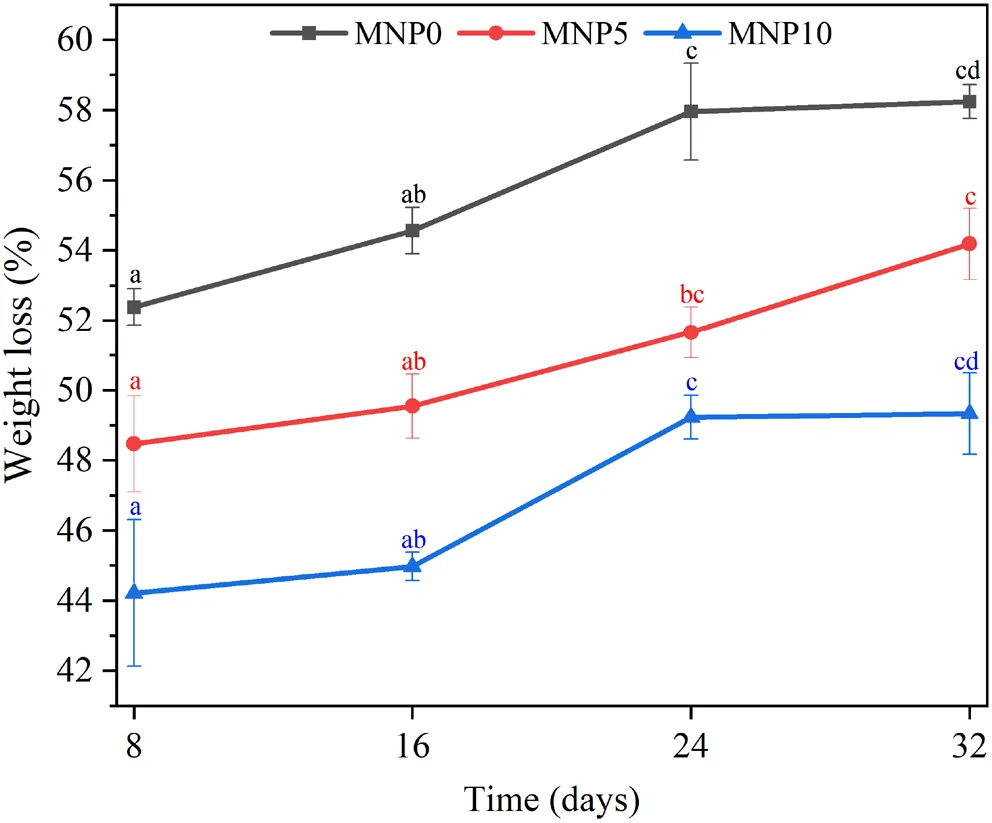
Weight loss of membranes immersed in PBS for 8, 16, 24, and 32 days. (Error bars followed by different letters on the same curve differ significantly by Tukey’s test at 5% probability).

Thus, the MNP0 and MNP10 membranes exhibited continuous mass loss during the first 24 days, followed by a tendency toward stabilization, suggesting a possible saturation of the hydrolytic degradation processes. In contrast, MNP5 demonstrated a distinct behavior, with no clear stabilization trend, maintaining a consistent degradation rate throughout the 32-day period. Between days 8 and 32, MNP5 experienced a mass loss variation of approximately 6.0%; however, no statistically significant difference was observed between the values at 24 and 32 days, suggesting a potential onset of stabilization.

It is also noteworthy that increasing the concentration of Manganite nanoparticles in the membrane composition led to enhanced resistance to degradation. This trend is particularly evident at the end of the experimental period, where MNP10 exhibited the lowest cumulative mass loss. Such behavior may be attributed to the structural reinforcement provided by the inorganic phase, which hinders polymer chain scission and slows down hydrolytic processes.

Furthermore, it is well established that the viscosity of the synthesis solution influences the kinetics of membrane formation. As the concentration of added nanoparticles increases, their tendency to aggregate can lead to the formation of multiple layers. In such cases, the cross-sectional structure becomes more flexible, while the surface becomes denser, thereby increasing the volume of internal pores.^69^

When correlating the mass loss of the membranes with their respective swelling degrees (Figure 8), an inversely proportional relationship is observed. The MNP0 retained the least amount of liquid and exhibited the greatest mass loss among all the membranes. Conversely, MNP10 demonstrated the highest swelling degree after 48 h and the lowest degradation rate. Therefore, in addition to enabling greater liquid retention, the MNP10 also proved to be more resistant to degradation compared to the other membrane compositions, not reaching 50% mass loss even after 32 days of testing.

## Water Vapor Permeability and Transmission Rate

The more cohesive the matrix of a polymeric structure, the lower its water vapor permeability (WVP) value.^48^ As shown in Figure 10a, increasing the concentration of nanoparticles in the polymeric matrix of the membranes led to a significant decrease in WVP values. This reduction is likely due to improved interactions among the membrane constituents, resulting in a more compact structure, as pointed out by SEM results (Figure 3). Thus, this structure has fewer pores, reducing the passage of water vapor through it.^70^

**Figure 10:**
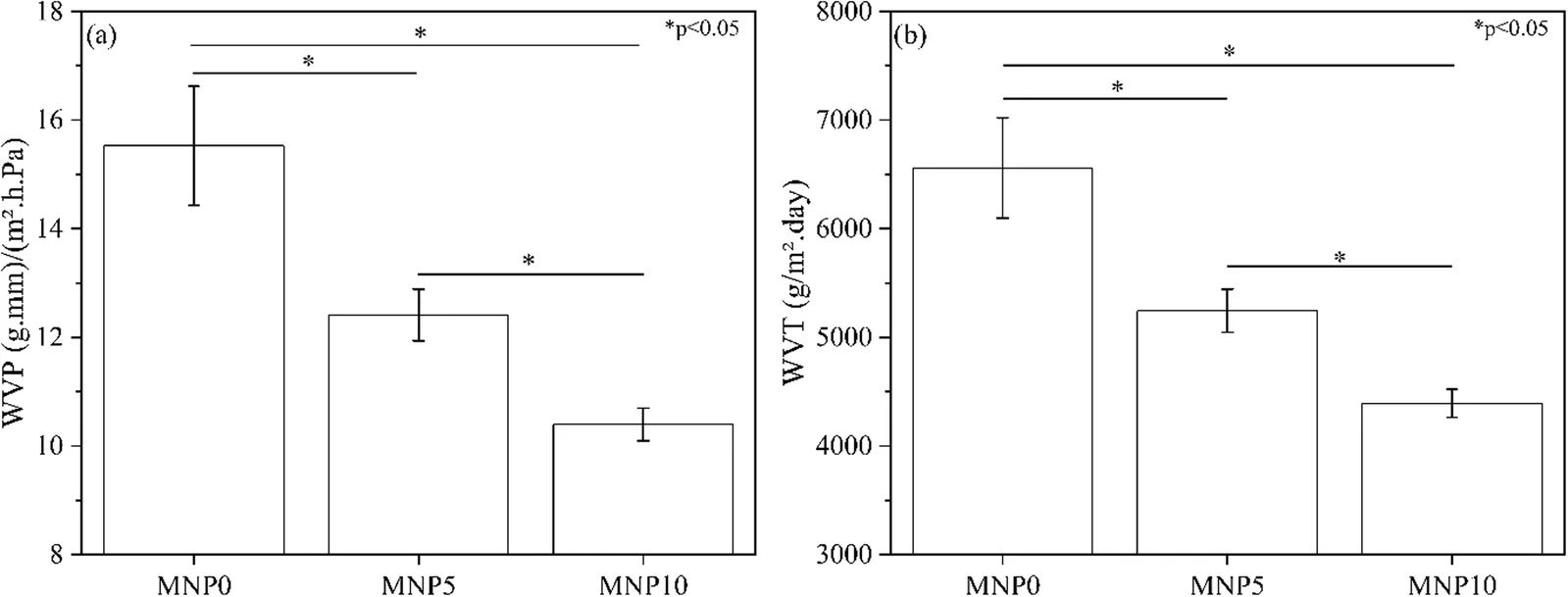
(a) WVP and (b) WVT of the membranes.

Based on the water vapor permeability (WVP) data of the membranes, the water vapor transmission rates (WVT) were calculated and are shown in Figure 10b. Analyzing the results for MNP0, MNP5, and MNP10, a significant reduction in WVT was observed as the concentration of Manganite in the polymer matrix increased, consistent with the WVP results. Although MNP10 exhibited the lowest permeability rate (Figure 10a), its WVT of 4,391 ± 128 g/m^2^.day was still more than twice the minimum recommended value in the literature, which is sufficient to maintain adequate moisture and function effectively as a wound dressing.

In fact, a WVT in the range of 2000 to 2500 g/m^2^·day is considered appropriate to maintain an ideal moist environment and prevent the accumulation of exudate on wounded skin. For reference, the WVT values for normal skin, skin with first-degree burns, and granulating wounds are approximately 200 ± 10, 280 ± 30, and 5100 ± 200 g/m^2^·day, respectively.^71,72^ Although MNP10 exhibited lower WVP values within the experimental group, it still presented high overall WVT rates, consistent with a porous and breathable hydro-matrix structure. This behavior is commonly observed in polysaccharide-based membranes, particularly starch systems, owing to their inherently hydrophilic nature and the absence of chemical modifications designed to enhance barrier performance, as reported by Vuillet et al.^22^

## Mechanical Properties Assessment by Tensile Testing

In tissue engineering, the successful application of a membrane requires consideration of the most common mechanical stresses encountered in dressings and skin adhesives.^43^ The results of the tensile strength tests for the membranes are presented in Figure 11.

**Figure 11:**
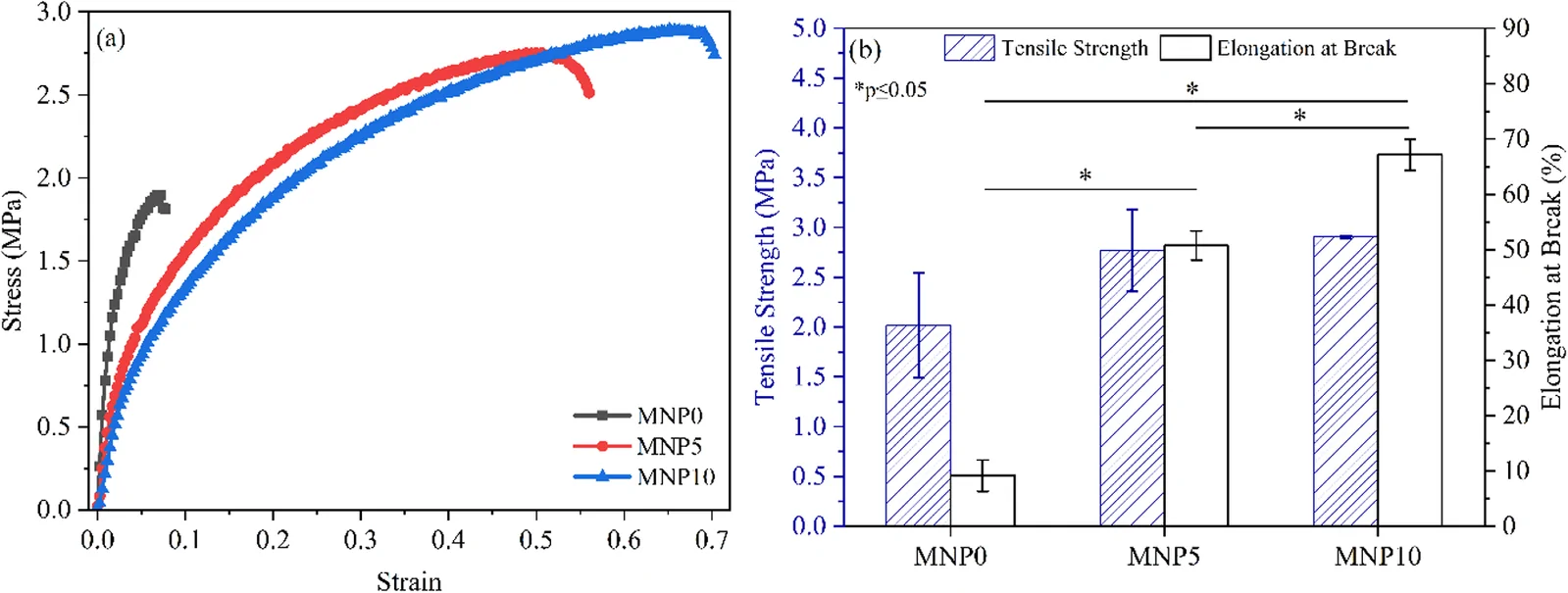
Mechanical properties of the membranes: (a) Stress–strain curves; (b) tensile strength and elongation at break.

The mechanical results indicate that increasing the concentration of Manganite in the membranes tends to enhance tensile strength and leads to a significant increase (*p* ≤ 0.05) in the elongation at break, as shown in Figure 11b.^73^ This behavior can be attributed to the effective dispersion of the nanoparticles and their interaction with the polymer’s functional groups, enabling stable incorporation into the polymer matrix.^74^ This incorporation weakens pre-existing intermolecular hydrogen bonds and promotes the formation of new hydrogen bonds between the polymer and the nanoparticles. Consequently, the mobility and rotation of polymer chains are facilitated, which enhances both tensile strength and elongation at break of the membranes.^75^ Moreover, the excellent mechanical performance and high surface area of the nanoparticles significantly contribute to the improved mechanical properties. Another contributing factor to the increased mechanical strength with higher Manganite concentration is the rise in the viscosity of the synthesis solution due to the presence of the nanoparticles.^76^

## Mechanical Properties Assessment by Nanoindentation

In addition to the mechanical properties obtained from tensile testing, the data for elastic modulus and hardness measured by nanoindentation are presented in Figure 12.

**Figure 12:**
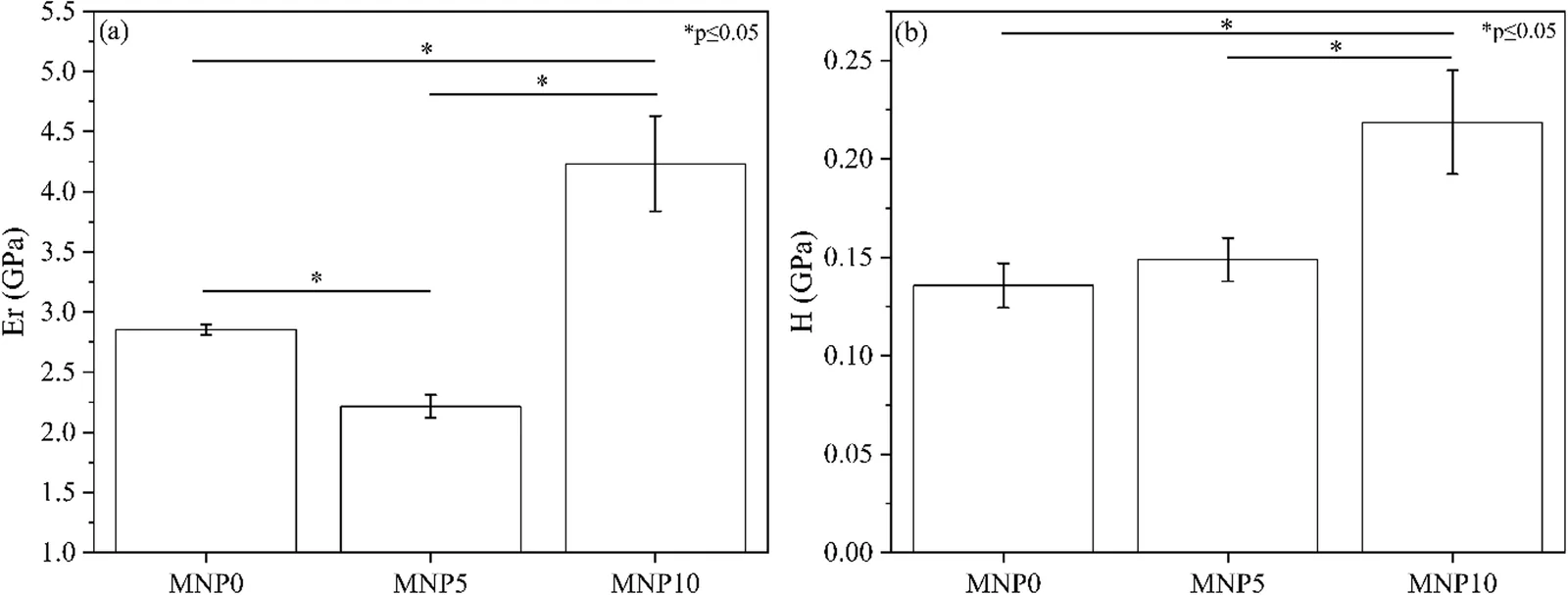
Mechanical properties of the membranes by nanoindentation: (a) Elastic modulus and (b) Hardness.

The increase in nanoparticle concentration significantly promotes greater stiffness for MNP10, as evidenced by higher values of both elastic modulus and hardness compared to the other membrane compositions (MNP0 and MNP5). These results corroborate the FTIR data, which indicate an increase in membrane density and in its rigidity as the nanoparticle content increases. However, while there was no statistically significant difference in hardness between MNP0 and MNP5, the latter showed a lower elastic modulus than the former. These variations in elastic modulus values may be due to sample inhomogeneity. Height variations caused by nanoparticle agglomeration on the membrane surfaces, as demonstrated by the roughness data and AFM images, can significantly affect nanoindentation measurements. Similar results were reported by Cai et al.^36^

Although nanoindentation data provide valuable information about the local mechanical reinforcement promoted by nanoparticle incorporation, the presence of localized ceramic nanoparticle domains indicates that these measurements mainly reflect the local surface mechanical response. In this context, the macroscopic tensile test results are more representative of the overall mechanical behavior and flexibility of the membranes.

## Magnetic Susceptibility Analysis

The magnetization curves of the Manganite powder and of the membranes containing these nanoparticles (MNP5 and MNP10) are shown in Figure 13. The pure La_0.4_Sr_0.6_MnO_3_ ceramic powder exhibited an “S” shaped hysteresis loop, indicating significant remanent magnetization.^32^ In contrast, the MNP5 and MNP10 membranes did not show this behavior, suggesting a strongly attenuated magnetic response with linear and reversible cycles — likely due to the low concentration of magnetic nanoparticles embedded in the polymer matrix.

**Figure 13:**
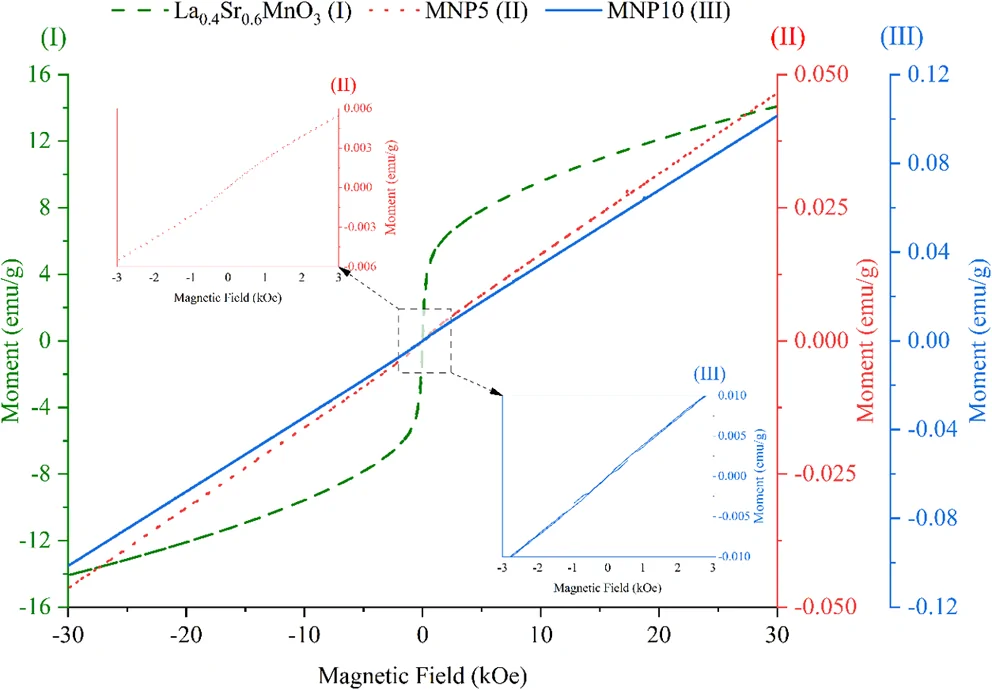
Variation of magnetization with the magnetic field for the La_0.4_Sr_0.6_MnO_3_ ceramic powder and the membranes: MNP5 and MNP10.

Although the MNP10 sample contains twice the ceramic nanoparticle content of MNP5, its magnetization was approximately 100 times lower than that of the isolated ceramic material. Nevertheless, an increase in the magnetic moment was observed with higher nanoparticle concentration, reaching approximately 0.05 emu/g for MNP5 and 0.10 emu/g for MNP10.

In addition, despite the lower concentration of nanoparticles incorporated into MNP5, a slight hysteresis loop was observed in its magnetic curves — a behavior not evident in the data for MNP10. This phenomenon may be associated with the formation of localized regions of nanoparticle agglomeration within the MNP5 polymer matrix, as evidenced by the AFM images. These agglomerated regions may have facilitated dipolar interactions between nanoparticles, enabling sufficient magnetic coupling to generate a detectable (albeit subtle) hysteretic response.

On the other hand, the absence of hysteresis in MNP10, even with a higher magnetic phase content, can be attributed to the more homogeneous dispersion of nanoparticles throughout its polymer matrix. This uniform distribution significantly reduces magnetic interactions between particles due to greater spatial separation, hindering the magnetic coupling necessary for the formation of stable magnetic domains and thereby preventing the emergence of a hysteresis loop, even at higher concentrations of magnetic material.^77,78^

This behavior reinforces the idea that, beyond the absolute quantity of magnetic phase, the morphology and distribution of nanoparticles within the matrix play a critical role in determining the final magnetic properties of the membranes. Compared to the behavior observed in the pure ceramic powder, where stronger magnetic coupling is favored by particle proximity and agglomeration, the limited interaction between nanoparticles dispersed in the polymeric medium appears to be decisive in suppressing the hysteretic response, particularly evident in MNP10.

## Antibacterial Susceptibility Test

The disk diffusion test was used to evaluate the antibacterial activity of the nanoparticles and membranes produced against the *Staphylococcus aureus* strain. For this purpose, a pure membrane (PM) — composed solely of babassu flour, glycerol, and water — and a 1.0% chlorhexidine digluconate (CD) solution were used as negative and positive controls, respectively. According to the data obtained, the PM did not exhibit any inhibition zone, whereas the CD exhibited an average inhibition halo of 4.6 ± 0.4 mm. Both showed statistically significant differences compared to the other samples tested against the*S. aureus*strain.

Analyzing the data presented in Figure 14, it was observed that, in addition to Manganite, antibacterial activity was also attributed to ascorbic acid, as inhibition halos were evident even in MNP0. Literature reports indicate that vitamin C effectively neutralizes *S. aureus* growth, exhibiting antibacterial activity. When combined with other antibacterial agents, such as antibiotics, it can enhance their effectiveness against resistant strains.^71,72^

**Figure 14:**
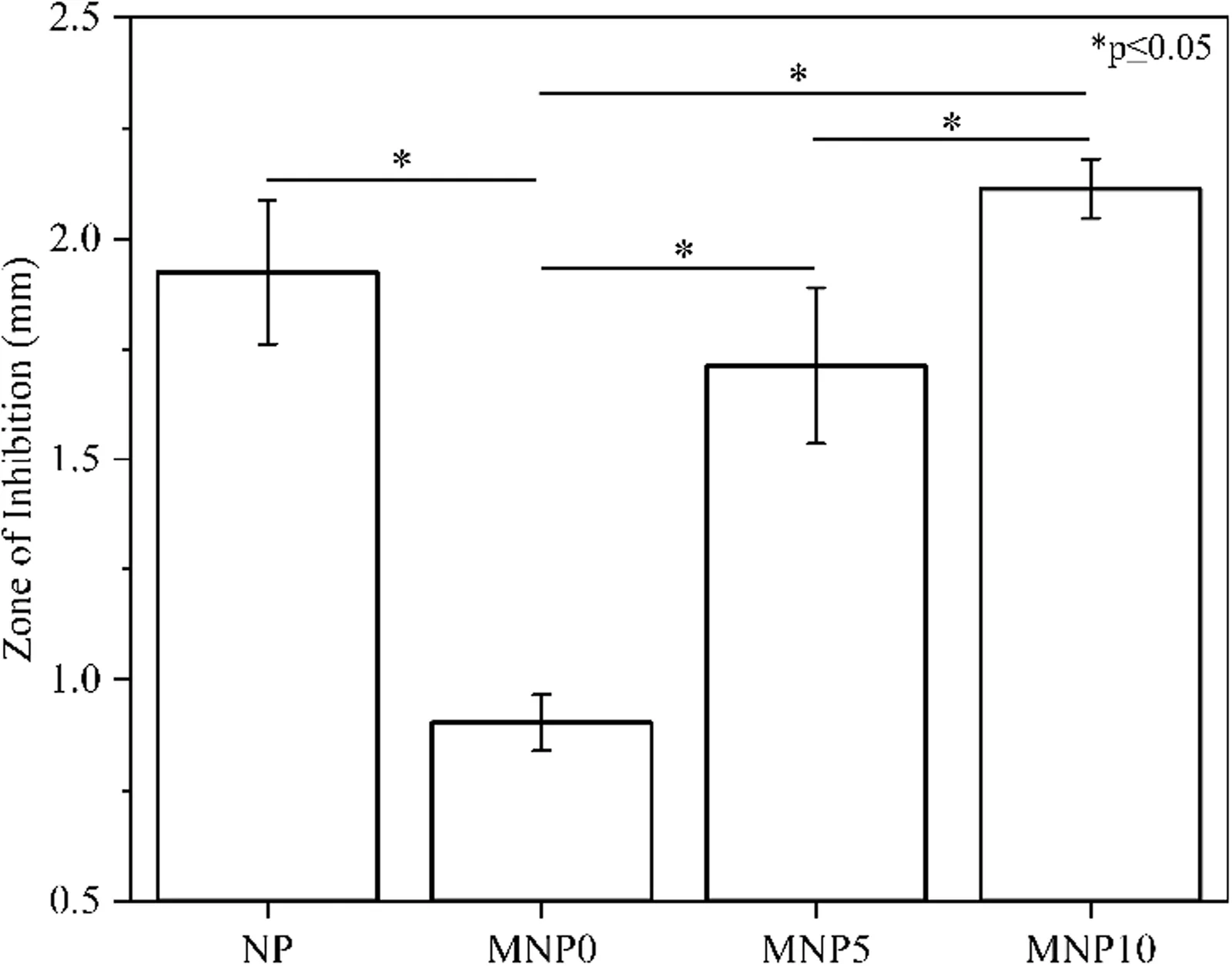
Dimensions of the inhibition zones for NP, MNP0, MNP5, and MNP10 against the *S. aureus* strain.

No significant differences were observed in the inhibition zones between NP (1.9 ± 0.2 mm) and MNP5 (1.7 ± 0.2 mm), as well as between NP and MNP10 (2.11 ± 0.07 mm). This indicates that the antibacterial properties of Manganite were retained when it was incorporated into the membranes at the analyzed concentrations. Thus, MNP10, which exhibited a significantly larger inhibition zone than MNP5 against the *S. aureus* strain, may be an effective strategy to inhibit the growth of this bacterium in wounds.

## Conclusion

Based on the characterization results of the membranes, the MNP10 demonstrated a more uniform distribution of nanoparticles on its surface, greater resistance to degradation in aqueous media, and the ability to retain and maintain a higher percentage of liquid for more extended periods. Additionally, it exhibited superior mechanical properties, with higher elongation at break (67 ± 3%), elastic modulus (4.2 ± 0.4 GPa), and nanohardness (0.22 ± 0.03 GPa) compared to the other membrane compositions.

However, despite the higher concentration of magnetic nanoparticles, MNP10, like the other membranes, did not exhibit sufficient magnetic susceptibility to produce a hysteresis loop under an applied electromagnetic field. This suggests that the nanoparticle concentration in the polymer matrix remains low, primarily due to limited physical interaction between well-dispersed particles within the matrix.

The antibacterial performance of MNP10 was comparable to that of pure nanoparticles and statistically superior to that of MNP0 and MNP5. Although MNP10 showed reduced permeability compared to the other membranes, its water vapor transmission rate (WVT) effectively meets the minimum requirement for maintaining a moist environment suitable for wound dressings. Therefore, a 10% (w/w) concentration of La_0.4_Sr_0.6_MnO_3_ in the membrane composition appears to be the most promising formulation for wound dressing applications based on the properties evaluated in this study. However, biological validation, particularly through *in vitro* cytotoxicity assays, is required before these membranes can be proposed for wound dressing applications.

Furthermore, future studies should investigate: (i) vitamin C stability, in addition to the identification of compounds released into PBS and their release kinetics, (ii) nanoparticle functionalization to improve dispersion in the polymeric matrix, and (iii) quantitative antibacterial activity against multiple bacterial strains. These investigations will provide a more comprehensive assessment of the membranes’ degradation behavior, biological performance, safety, and therapeutic potential.

## Data Availability

Data will be made available on request.
